# Stress, Subordination, and Anomalies of Feeding Across the Tree of Life: Implications for Interpreting Human Eating Disorders

**DOI:** 10.3389/fpsyg.2021.727554

**Published:** 2021-10-05

**Authors:** B. Natterson-Horowitz, Julia H. Cho

**Affiliations:** ^1^Division of Cardiology, David Geffen School of Medicine at UCLA, Los Angeles, CA, United States; ^2^Department of Human Evolutionary Biology, Harvard University, Boston, MA, United States; ^3^Harvard Medical School, Boston, MA, United States

**Keywords:** social subordination, hypophagia, hyperphagia, eating disorders, animal models

## Abstract

Eating behaviors of animals living in naturalistic environments offer unique insights into several dysregulated eating patterns observed in humans. Social subordination is a known precipitant of hyperphagia and hypophagia in human beings, and examples of similar responses have been identified in a phylogenetically widespread range of vertebral species. This points to potentially conserved, patterned responses to animals navigating lives within social hierarchies. Self-imposed food restriction in subordinate fish and hyperphagic responses in socially subordinated bird and primate individuals may represent evolved adaptations to the stress of social subordination. As such, hyperphagic and hypophagic responses to social subordination in these species may model the natural history, neurobiology, and behavioral ecology of human dieting and bingeing more accurately than some current animal models.

Phylogenetically widespread similarities in eating patterns under the stress of social subordination point to potentially shared biological benefits of these behaviors across species and the role of evolutionary trade-offs, adaptations, and other processes in shaping them. The application of a broadly comparative lens to disordered eating behaviors in other species exposes important similarities and differences between neurophysiology of eating across species. In doing so, it highlights the value of phylogenetic analyses and macroevolution as tools for identifying novel, naturally occurring models for understanding disordered human eating. Moreover, this approach introduces the intriguing possibility that human cultural influences on disordered eating may have far more ancient origins than previously considered.

## Introduction

### Hierarchies Impact Eating Behavior

Social hierarchies can be found across the animal kingdom, from insects (Monnin and Peeters, 1999; Huisken et al., 2021) and crustaceans (Edwards et al., 2003; Stewart and Tabak, 2011) to fish (Olsen and Ringø, 1999), birds (Noble, 1939), and primates (Michopoulos et al., 2012). While these vary significantly in their structure and flexibility, hierarchies emerge spontaneously across a phylogenetically wide range of social species. The precise evolutionary function of these hierarchies remains the subject of ongoing debate. However, across the diversity of hierarchies found among animal groups, a phylogenetically widespread linkage can be found between patterned eating behaviors and an individual’s position within its social hierarchy in what appear to be widely conserved patterned eating responses.

Attaining a higher position in a hierarchy, in general, leads to greater access to food (Lee et al., 2018). Since larger size is associated with dominance in many animal hierarchies, a dominant’s greater access to food leads to larger size. Increased nutrition coupled with greater access to mating opportunities leads to greater reproductive output and overall fitness (Paull et al., 2010). Individuals who are subordinate to dominants generally have less access to (high quality) food resources, with dominant animals commonly restricting subordinates’ access to food (Ekman and Lilliendahl, 1993; Ang and Manica, 2010). This further reinforces the size and therefore power gradient within the hierarchy and reduces the fitness of subordinates.

Evidence links lower social status to poorer health outcomes in both human and primate societies (Segerstrom and Miller, 2004; Sapolsky, 2005). Animals of lower rank in stable hierarchies also experience more stress from insufficient resources, dominant aggression, and limited opportunities to mate (Blanchard et al., 2001; Sapolsky, 2005; Filby et al., 2010). It is not surprising, therefore, that animals in subordinate positions might exhibit patterned eating behaviors such as bingeing or restricting food as adaptations to physical challenges associated with their low social status (Koebele, 1985; Cruz et al., 2007; Woog et al., 2012; Obirikorang et al., 2020). Given the conserved neurobiology that controls hierarchy development and thus influences appetite and feeding, we posit that the studies of socially stressed animals partaking in abnormal feeding behaviors may have mechanistic or clinical pearls to offer to human psychiatric medicine and eating disorder care.

**Figure 1:**
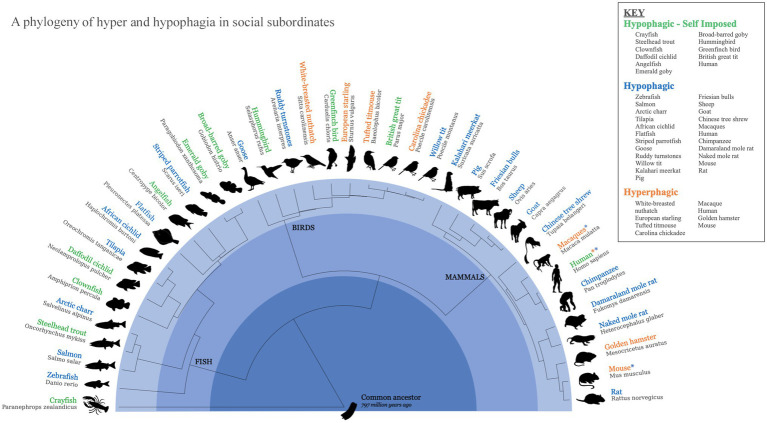
Animal models for hypophagia and hyperphagia in socially subordinated animals. A phylogeny of the potential naturally occurring animal models for hyperphagic and hypophagic responses to social stress. References: Hypophagia - Self Imposed (Abbott and Dill, 1989; Ekman and Hake, 1990; Hiebert, 1991; Gosler et al., 1995; Buston, 2003; Heg et al., 2004; Ball and Crawford, 2005; Sloman et al., 2005; Munday et al., 2006; Wilson et al., 2008; Wong et al., 2008; Moore et al., 2013); Hypophagia (Purdom, 1974; Jobling and Wandsvik, 1983; Clifton, 1990; Owen, 1990; Ekman and Lilliendahl, 1993; Webb, 1993; Hofmann et al., 1999; Barroso et al., 2000; Maclean and Metcalfe, 2001; Wittig and Boesch, 2003; Bartolomucci et al., 2004; Ball and Crawford, 2005; Vahl et al., 2005; Dengler-Crish and Catania, 2007; Partida et al., 2007; Wilson et al., 2008; Choi and Kim, 2010; Filby et al., 2010; Young and Bennett, 2010; Michopoulos et al., 2012; Woog et al., 2012; Moore et al., 2013; Wang et al., 2013; Treasure et al., 2015; Dubuc and Clutton-Brock, 2019; Schalla and Stengel, 2019; Carneiro-Nascimento et al., 2020; Obirikorang et al., 2020); Hyperphagia (Witter and Swaddle, 1995; Pravosudov et al., 1999; Bartolomucci et al., 2004; Ball and Crawford, 2005; Foster et al., 2006; Razzoli et al., 2015; Brownley et al., 2016; Carneiro-Nascimento et al., 2020).

Subordinate animals, whether due to food insecurity or other stressors associated with their position, exhibit different patterns of eating than dominants. As shown in Figure 1, subordinates across a phylogenetically wide range of species respond with several patterned responses to these challenges through behavioral adaptations in their eating: Both hyperphagic (Witter and Cuthill, 1993; Witter and Swaddle, 1995; Pravosudov et al., 1999; Bartolomucci et al., 2004; Foster et al., 2006; Michopoulos et al., 2012) and hypophagic (Purdom, 1974; Jobling and Wandsvik, 1983; Koebele, 1985; Clifton, 1990; Barroso et al., 2000; Sloman et al., 2000; Maclean and Metcalfe, 2001; Wittig and Boesch, 2003; Earley et al., 2004; Sloman et al., 2005; Tamashiro et al., 2005; Vahl et al., 2005; Munday et al., 2006; Cruz et al., 2007; Dengler-Crish and Catania, 2007; Partida et al., 2007; Filby et al., 2010; Young and Bennett, 2010; Woog et al., 2012; Wang et al., 2013; Dubuc and Clutton-Brock, 2019; Carneiro-Nascimento et al., 2020; Obirikorang et al., 2020) responses to social subordination have been observed in mammals, birds, fish, and invertebrates. While the number of species in which these responses have been studied is not large, the phylogenetic diversity of species in which these eating responses have been recorded, coupled with a conserved neurophysiology, argues for an ancient linkage between behavioral responses to social subordination in humans and other animals. In species from crawfish and Arctic charr to woodland birds and rodents, status descent has been shown to activate similarly patterned behavioral responses which emerge from what appears to be the induction of neurophysiological systems conserved across chordates (Jobling and Wandsvik, 1983; Pravosudov et al., 1999; Bartolomucci et al., 2004; Maniscalco et al., 2013; Carneiro-Nascimento et al., 2020).

Underlying the connections between social position and eating behavior is the widely conserved ability of social species to detect shifts in their social status (Chiao, 2010). Social behavioral networks signal shifts in position through nonverbal, implicit signals which, in humans, are interpreted by the inferior parietal lobe, dorsolateral, and ventrolateral prefrontal cortices, and portions of the occipitotemporal lobe (Chiao, 2010). Recognition of status descent activates a suite of subordinate behaviors including characteristic eating responses.

In naturalistic settings, animal eating is impacted by both predatory and social stressors with both hyperphagia and hypophagia emerging in response to both (Choi and Kim, 2010; Maniscalco et al., 2013). Focusing on socially shaped eating behaviors provides a window into the ancient origins of common hyperphagic and hypophagic responses to social stress and status descent among subordinate animals.

## Stress and Eating Patterns in Wild Animals

Predatory stress is well known to shape the timing, quantity, duration, and location of eating in wild animals (Choi and Kim, 2010; Natterson-Horowitz and Bowers, 2013). Social stress, too, affects appetite and eating (Razzoli et al., 2015). Social position within hierarchies is a source of stress for mammals, birds, and fish living within them (Hobson et al., 2021). Position in hierarchy generally shapes an individual’s access to food, with higher ranking individuals controlling and consuming critical resources, and subordinates facing an increased risk of starvation and death (Vahl et al., 2005; Cruz et al., 2007; Lee et al., 2018; Dubuc and Clutton-Brock, 2019). This reality reinforces the fitness-reducing impact of subordinate status within animal groups and underscores the necessity of patterned eating behaviors to respond to these challenges.

### Hyperphagia in Subordinate Animals

Chronic stress elicits a range of eating responses in humans, with approximately half increasing and half decreasing their food intake (Torres and Nowson, 2007; Macht, 2008). A series of independent factors are associated with the tendency toward hypo- or hyperphagia in response to chronic stress. These include stress type and severity, arousal level, and food options available (Macht, 2008; Rutters et al., 2009). Chronic stress is known to raise glucocorticoid levels, which can lead to increased appetite (Dallman, 2010; Sominsky and Spencer, 2014). The release of central endogenous opioid peptides in association with overeating and hyperphagia may be a soothing counter-response to the noxious experience of rising glucocorticoids (Mercer and Holder, 1997; Dallman et al., 2003).

Hyperphagic responses to social stress have been identified in many other species. Michopoulos et al. found that subordinate rhesus monkeys consumed significantly more calories than dominants, preferring high fat-high sugar diets over low fat-high fiber diets (Michopoulos et al., 2012). Male Syrian hamsters, when repeatedly forced into social defeat by a conspecific dominant, significantly increase their food intake and body mass relative to control hamsters (Foster et al., 2006).

Similarly, subordinate woodland birds carry greater fat reserves than dominants do (Witter and Swaddle, 1995; Pravosudov et al., 1999). While lower energy reserves for dominant birds may seem paradoxical, greater mass reduces maneuverability when evading predators (Witter and Cuthill, 1993). Dominant birds are able to maintain a lower body mass, thus protecting them from predation, because of better access to food and lower risk of starvation.

In a 2004 experiment, subordinate mice were subjected to chronic social stress in the form of repeated attacks from a dominant mouse (Bartolomucci et al., 2004). Despite no changes in food intake, all subordinates exhibited increased body weight at the end of the experiment, implying changes in metabolic functions aimed at storing more energy (Bartolomucci et al., 2004). A similar study in mice found that subordinate mice experienced reduced satiety than control mice despite increased hyperphagia and overall consumption, suggesting additional neuroendocrine causes of subordinate overeating (Maniscalco et al., 2013).

### Hypophagia in Subordinate Animals

The smaller body size typical of subordinate animals has traditionally been attributed to their restricted food access due to dominant control or aggression (Lee et al., 2018). Subordinate animals eat less, often because dominants simply will not permit them to consume available food resources (Munday et al., 2006; Choi and Kim, 2010; Hobson et al., 2021). Their lower consumption of food contributes to their lower rate of growth. Notably, even when dominants may not directly intimidate subordinates around food access, hypophagia and decreased growth are noted. For example, isolated flatfish grown in separate containers displayed a negative correlation between size and growth rate – the smaller fish grew faster even when fed the same amount as the larger fish (Purdom, 1974). Yet, when small and large fish were reared together, allowing a size-based social hierarchy to form, Purdom found the opposite: the smaller, and therefore more subordinate fish, grew slower than the larger, more dominant fish (Purdom, 1974).

Such growth suppression of smaller, low-ranking animals has been observed in numerous other fish (Koebele, 1985; Clifton, 1990; Hofmann et al., 1999; Maclean and Metcalfe, 2001; Munday et al., 2006; Cruz et al., 2007; Filby et al., 2010; Woog et al., 2012; Obirikorang et al., 2020), birds (Vahl et al., 2005; Partida et al., 2007), mammals (Barroso et al., 2000; Tamashiro et al., 2005; Dengler-Crish and Catania, 2007; Young and Bennett, 2010; Wang et al., 2013; Dubuc and Clutton-Brock, 2019; Carneiro-Nascimento et al., 2020), and also primates (Wittig and Boesch, 2003). Numerous mechanisms have been suggested or observed to contribute to this phenomenon: monopoly of food resources and intimidation by dominants (Jobling and Wandsvik, 1983; Maclean and Metcalfe, 2001; Cruz et al., 2007); hormonal changes affecting appetite, growth rate, digestive tract function, and metabolic rate (Jobling and Wandsvik, 1983; Sloman et al., 2000, 2005; Filby et al., 2010); and changes in activity level and foraging behaviors (Earley et al., 2004).

Subordinate undereating in agricultural animals has also been identified as a possible model for understanding disordered food restriction behavior in humans (Treasure and Owen, 1997). In wasting pig syndrome (e.g., thin sow syndrome), social stressors including bullying and premature separation from mothers may induce undereating in subordinates (MacLean, 1968). In some severe cases, the self-induced starvation may trigger hypothalamic and pituitary changes like those seen in humans with anorexia nervosa (MacLean, 1968). Similarities in undereating behavior among subordinate pigs, sheep, goats, and rats points to a potential shared underlying mechanism among mammals (Owen, 1990; Webb, 1993; Treasure and Owen, 1997; Schalla and Stengel, 2019). While thin sow syndrome and undereating behaviors in other species do not represent perfect models for anorexia nervosa or other human eating disorders, there are significant parallels in phenomenology and mechanism.

### Self-Imposed Hypophagia in Subordinate Animals

In some species, subordinate animals have been observed to eat less than dominants even when the dominants are not aggressive or limit subordinate access to food. This subordinate “dieting” behavior, as the observing biologists describe it, appears to be a strategy to stay smaller than a size that will attract dominant aggression or even lead to eviction from the safety of the group (Abbott and Dill, 1989; Buston, 2003; Heg et al., 2004; Wong et al., 2008; Ang and Manica, 2010; Stewart and Tabak, 2011).

To understand why subordinates appeared to be self-restricting their food consumption in the absence of direct threat, Wong et al. studied the eating behavior of the *Paragobiodon xanthosomus* (Wong et al., 2008). The social hierarchy of these coral reef-living goby is a reproductive and size-based queue, with the dominant, breeding male and female at the top and several subordinate, non-breeding females below. When the dominant female dies, the highest ranking subordinate grows to fill its spot and can now procreate; all the subordinates below it also move up in rank and grow accordingly larger (Wong et al., 2008).

In their experiment, Wong et al. fed supplemental food to experimental Rank 4 fish and observed that they grew at a faster rate than their immediately dominant Rank 3 fish, as well as Rank 4 fish from control groups (Wong et al., 2008). Yet, about half of the experimental Rank 4 fish soon stopped eating the supplemental food, despite no interference from dominant fish. When measured, it was found that Rank 4 fish stopped eating when they reached 90–95% of the body size of the Rank 3 above it. The other half of the Rank 4s that did not stop eating and grew larger than 95% of the Rank 3’s body size was promptly evicted from the group by more dominant fish (Wong et al., 2008). There was no statistically significant difference in aggression from Rank 3s to Rank 4s between the experimental and control groups, removing dominant aggression as a confounding factor that could have caused the self-restriction in food intake (Wong et al., 2008).

The authors called this strategic undereating behavior “dieting” because the self-imposed food restriction appeared to be a strategic approach of preventing an increase in body size in order to avoid detection and eviction by a dominant (Wong et al., 2008).

This behavior has been described in a relatively small number of animals, limited mostly to various species of fish and birds (Abbott and Dill, 1989; Ekman and Hake, 1990; Hiebert, 1991; Ekman and Lilliendahl, 1993; Gosler et al., 1995; Buston, 2003; Heg et al., 2004; Ang and Manica, 2010; Stewart and Tabak, 2011). However, given the absence of research into animal eating patterns through a comparative lens, it is not unreasonable to conclude that the behavior could extend beyond the species identified. We predict that other group-living animals including reptiles, mammals, and nonhuman primates may also share neurobiology promoting strategic undereating under specific social conditions.

Food restriction aimed at maintaining a desired body size might seem to be a uniquely human phenomenon, with significant links to contemporary cultural and other factors associated with modern human life (Putterman and Linden, 2004). Yet, this very assumption may underlie the limited comparative research into this connection. The conserved nature of interdependence of brain systems underlying appetite, foraging, and the navigation of social hierarchies in vertebral taxa from crustaceans to mammals suggests that the behavioral responses of wild animals under social stress remain unexplored sources of insight into human eating. A phylogenetically broad scope has the potential to uncover new animal models for studying eating pathology and uncovering the roots of altered eating behavior under the stress of social stress in humans.

## Discussion

### Naturally Occurring Animal Models

Efforts to understand dysregulated human eating usually focus on traditional animal models including zebrafish, rodents, and some invertebrate species (Casper et al., 2008). Reductive investigation has identified a range of neurophysiologic processes associated with disordered human eating. However, these mechanistic explanations fail to provide a broader, evolutionary explanation for eating behavior which, in our species, may threaten health and life.

While the eating behavior of laboratory animals from zebrafish and rodents to selected invertebrates provides some insights, patterns in 5.5 million other animal species on Earth remain an underexamined source of insights. Understanding the factors which shape the eating behavior of wild animals provides a window into the evolutionary origins of human eating. Specifically, it casts focus on the adaptive (fitness-enhancing) properties of hypophagia and hyperphagia – behaviors which, in humans, are generally detrimental (Neumark-Sztainer et al., 2011; Liechty and Lee, 2013).

### Shared Mechanisms

An important question raised by the identification of overeating and undereating in the wild is whether and to what degree these mechanisms are present in modern humans. Several strategies can be used to answer this question. Among the simplest are basic phylogenetic analyses which can reveal the presence or absence of connection across gene ontologies relevant to disordered human eating. The extent to which the eating responses seen in fish and other animal subordinates have salience for human eating disorders is linked to the degree to which neuroanatomical and neurophysiologic systems are conserved across species. Significant functional similarities in certain neuroanatomical neurophysiological systems related to appetite, eating, and social flux can be found across chordates. In mammals, the substantia nigra (SN) extends dopaminergic projections into the dorsal striatum/caudoputamen (CPu) in order to stimulate interest in feeding (Soengas et al., 2018). In fact, dopamine-deficient mice were shown to become aphagic and eventually starve to death (Zhou and Palmiter, 1995; Palmiter, 2007), and feeding behavior could be rescued by administering dopamine to the SN-CPu pathway (Szczypka et al., 2001). In fish, the nucleus of the posterior tubercle appears homologous to the SN (Meredith and Smeets, 1987), and a system of dopaminergic neurons connected to the CPu has been identified but their relationship to reward and feeding has not yet been assessed (Rink and Wullimann, 2001). The mammalian arcuate nucleus (ARC), a collection of neurons in the hypothalamus, plays a key role by secreting the orexigenic hormone NPY and regulating anorexigenic CART/POMC nuclei, as well as being the only neurons to secrete the orexigenic hormone agouti-related peptide (AgRP) (Bagnol et al., 1999; Cerdá-Reverter et al., 2000; Waterson and Horvath, 2015; Soengas et al., 2018). In fish, only the lateral tuberal nucleus (NLT) and dorsal hypothalamus (Hd) secrete AgRP, connecting these regions to mammalian ARC (Agulleiro et al., 2014; Soengas et al., 2018). The secretion of NPY by fish NLT further strengthens homologies in appetite (Hobson et al., 2021).

The identification of shared gene ontologies and neurobiological pathways connecting patterned eating behaviors to life history characteristics of animals should provide important reassurance that despite the phylogenetic distance between fish and modern humans, deeply conserved biological systems link behavior across species.

### Evolutionary Perspectives: The Adaptive Value of Hypophagic and Hyperphagic Behavior

In 1963, Nikolaas Tinbergen, an animal behaviorist who won the Nobel Prize in medicine a decade later, identified the limitations of mechanistic or proximate explanations (Tinbergen, 1963). Reductive insights, he explained, were essentially descriptions of “how” rather than “why” a behavior existed in an animal species. To fully understand “why” required a phylogenetic investigation to determine how the behavior might enhance survival and/or reproduction in the wild. The application of a Tinbergen lens to disordered eating in humans reveals insights that more anthropocentric and reductive investigations cannot.

Classically subordinate animals weigh less because dominants control food resources, at times restricting subordinate access to them. In some taxa, this suppresses reproductive function of subordinates, providing a fitness advantage to dominant individuals (Dengler-Crish and Catania, 2007; Young and Bennett, 2010; Dubuc and Clutton-Brock, 2019). Yet, Wong et al. found that some subordinates may self-restrict, despite access to food and absence of aggression from dominants (Wong et al., 2008). Various fitness benefits associated with a desired body size may explain hypophagia in subordinate animals. For example, chronic calorie restriction is associated with longer lifespan in a wide range of species (Pifferi et al., 2018). An additional adaptive hypothesis for self-restriction may be that a “slow” life history strategy may be the most fitness-enhancing response for subordinates. Whatever the adaptive nature of self-restriction hypophagia, the existence of this patterned response to social subordination offers insights that phylogenetically narrow perspectives cannot.

Overeating in response to social stress is common among humans and various animal species. Notably, while increased food consumption and greater body weight generally support dominant status and increase survival and reproductive success, in some species, this is not the case. Here again, a broad comparative analysis may shed light on some of the factors promoting stress eating in humans. For example, birds tend to “travel light,” in that they only eat what is necessary because increasing body mass will decrease maneuverability when escaping from predators (Witter and Cuthill, 1993). Despite this, Pravosudov et al. showed that subordinate members of several woodland birds overconsumed whenever food was available to combat the increased starvation risk for subordinate versus dominant birds during the wintertime (Pravosudov et al., 1999). Rhesus monkeys also display subordinate overconsumption relative to dominants in settings of social stress (Michopoulos et al., 2012). The conserved neurobiology of stress and eating across species points to potential novel animal models for understanding stress-induced under and overeating.

## Conclusion

For hundreds of millions of years, social animals have been faced with the parallel and interconnected tasks of finding food while navigating consequential social hierarchies. It is therefore not surprising that the neurophysiological systems associated with these challenges would be highly conserved and present in modern humans. This knowledge has the potential to not only expose novel animal models for eating disorders, but to provide an expanded, evolutionarily and ecologically informed understanding of the causes of disordered human eating.

Notably, the range of species with vulnerability to these behaviors is likely larger than what is presented in this paper. Long-standing anthropocentrism in the fields of human medicine and psychiatry has limited the study of animal eating behaviors and their use as a novel approach for investigation. Thus, the existence of hyperphagic and hypophagic responses to social stress in mammals, birds, fish, and invertebrates disrupts the conventional paradigm implicating human-specific contemporary cultural forces and social pressures as the primary “causes” of eating disorders. In fact, the naturalistic occurrence of self-restricted food intake or episodes of hyperphagia in other species, as well as phylogenetically widespread neuroanatomic, physiologic, functional, and evolutionary factors, point to a far more ancient theory rooted in evolutionary trade-offs, adaptations, and fitness. In addition, the spontaneous occurrence of “dieting” and “bingeing” in natural settings underscores the important role of ecological factors in triggering, promoting, and suppressing these behaviors in humans. Lastly, we hope that deeper investigation into the ancient, widespread origins of over and undereating in humans may provide some relief from the shame and stigmatization surrounding eating disorders which cause suffering and may even impede progress in understanding these behaviors.

## Data Availability Statement

The original contributions presented in the study are included in the article/supplementary material, further inquiries can be directed to the corresponding author.

## Author Contributions

Both authors are co-first authors. BNH and JC contributed to conception, design, primary research, figure development, and writing of the manuscript. All authors contributed to the article and approved the submitted version.

## Conflict of Interest

The authors declare that the research was conducted in the absence of any commercial or financial relationships that could be construed as a potential conflict of interest.

## Publisher’s Note

All claims expressed in this article are solely those of the authors and do not necessarily represent those of their affiliated organizations, or those of the publisher, the editors and the reviewers. Any product that may be evaluated in this article, or claim that may be made by its manufacturer, is not guaranteed or endorsed by the publisher.
